# Exploiting RIG-I-like receptor pathway for cancer immunotherapy

**DOI:** 10.1186/s13045-023-01405-9

**Published:** 2023-02-08

**Authors:** Yangfu Jiang, Hongying Zhang, Jiao Wang, Jinzhu Chen, Zeyu Guo, Yongliang Liu, Hui Hua

**Affiliations:** 1 Laboratory of Oncogene, State Key Laboratory of Biotherapy, West China Hospital, Sichuan University, Chengdu, 610041 China; 2 School of Basic Medicine, Chengdu University of Traditional Chinese Medicine, Chengdu, 610075 China; 3 Laboratory of Stem Cell Biology, West China Hospital, Sichuan University, Chengdu, 610041 China

**Keywords:** Cancer, Immunotherapy, Oncolytic virus, RIG-I, RIG-I-like receptors, RNA therapy, Viral mimicry

## Abstract

RIG-I-like receptors (RLRs) are intracellular pattern recognition receptors that detect viral or bacterial infection and induce host innate immune responses. The RLRs family comprises retinoic acid-inducible gene 1 (RIG-I), melanoma differentiation-associated gene 5 (MDA5) and laboratory of genetics and physiology 2 (LGP2) that have distinctive features. These receptors not only recognize RNA intermediates from viruses and bacteria, but also interact with endogenous RNA such as the mislocalized mitochondrial RNA, the aberrantly reactivated repetitive or transposable elements in the human genome. Evasion of RLRs-mediated immune response may lead to sustained infection, defective host immunity and carcinogenesis. Therapeutic targeting RLRs may not only provoke anti-infection effects, but also induce anticancer immunity or sensitize “immune-cold” tumors to immune checkpoint blockade. In this review, we summarize the current knowledge of RLRs signaling and discuss the rationale for therapeutic targeting RLRs in cancer. We describe how RLRs can be activated by synthetic RNA, oncolytic viruses, viral mimicry and radio-chemotherapy, and how the RNA agonists of RLRs can be systemically delivered in vivo. The integration of RLRs agonism with RNA interference or CAR-T cells provides new dimensions that complement cancer immunotherapy. Moreover, we update the progress of recent clinical trials for cancer therapy involving RLRs activation and immune modulation. Further studies of the mechanisms underlying RLRs signaling will shed new light on the development of cancer therapeutics. Manipulation of RLRs signaling represents an opportunity for clinically relevant cancer therapy. Addressing the challenges in this field will help develop future generations of cancer immunotherapy.

## Introduction

Chronic infection and inflammation are established risk factors for carcinogenesis. A substantial proportion of human cancers is attributable to chronic infection with Helicobacter pylori (H. Pylori), hepatitis B virus (HBV), hepatitis C virus (HCV), Epstein–Barr virus (EBV), Kaposi sarcoma-associated herpesvirus and human papillomavirus (HPV) [1–3]. Worldwide, the most prevalent microorganisms attributable to cancer incidence are H. pylori, HPV and HBV/HCV, which are most associated with gastric, cervical and liver cancer, respectively [4]. Mechanistically, sustained inflammation or immune response during host–pathogen interaction increases cancer risk by promoting mutagenesis, genome instability, epigenetic changes and cytokine response. Meanwhile, viral and bacterial proteins can directly induce oncogenic signaling, thereby promoting tumorigenesis [5]. These effects not only impact parenchymal cells that subsequently transform into malignant cells, but also reprogram stromal cells such as macrophages and fibroblasts, creating a permissive tumor microenvironment. Chronic inflammation may lead to tissue injury and excessive accumulation of extracellular matrix that has complex roles in tumorigenesis [6]. While intracellular H. pylori infection is a well-established risk factor for gastric cancer, recent studies also indicate the association of bacterial infection with other tumor types, such as lung, pancreatic and colorectal cancer [7–12].

Following pathogen infection, a local or systemic immune response is initiated to attenuate the infection. On the other hand, pathogens may evade the host immune responses and induce immunosuppression, leading to chronic inflammation. The host–pathogen interactions are involved in different stages of pathogen infection. Pathogen-associated molecular patterns (PAMPs) and damage-associated molecular patterns (DAMPs) turn on pattern-recognition receptors (PRRs) such as Toll-like receptors (TLRs), cyclic GMP-AMP synthase (cGAS)-stimulator of interferon response CGAMP interactor (STING) and nucleotide-binding oligomerization domain-containing protein 2 (NOD2), leading to the increased assembly of an innate immune complex termed inflammasome that contains Nod-like receptor family pyrin domain-containing protein (NLRP), NLRC4 or absent in melanoma 2 (AIM2)-like receptors, caspase-1 and pro-IL-1β [13]. Activation of inflammasomes eventually triggers caspase-1-dependent release of the proinflammatory cytokines IL-1β and IL-18 and induces pyroptotic cell death in a gasdermin-dependent manner [14]. Excessive or repeated inflammasome activation underlies the pathology of inflammatory diseases, tissue damage and carcinogenesis. Due to the roles of TLR and STING signaling in immune responses such as tumor antigen presentation, T cell recruiting chemokines secretion within the tumor, and inflammation within the tumor microenvironment that supports cytotoxic immune cell function, many TLR and STING agonists have been developed to serve as vaccine adjuvants or cancer immunotherapeutics [15]. The lipid A subunit of bacterial lipopolysaccharide, a TLR4 agonist, is an adjuvant for the US Food and Drug Association (FDA)-approved HPV vaccine Cervarix® that shows high efficacy against cervical cancer [16, 17]. In addition, the TLR7/8 activator Imiquimod has been used to treat basal cell carcinoma [18]. However, activation of tumor cell TLRs may also promote tumor cell proliferation and invasion, resistance to apoptosis, and immune evasion [15]. These hurdles need to be overcome for realizing the antitumor potential of TLR agonists in the clinic.

**Fig. 1:**
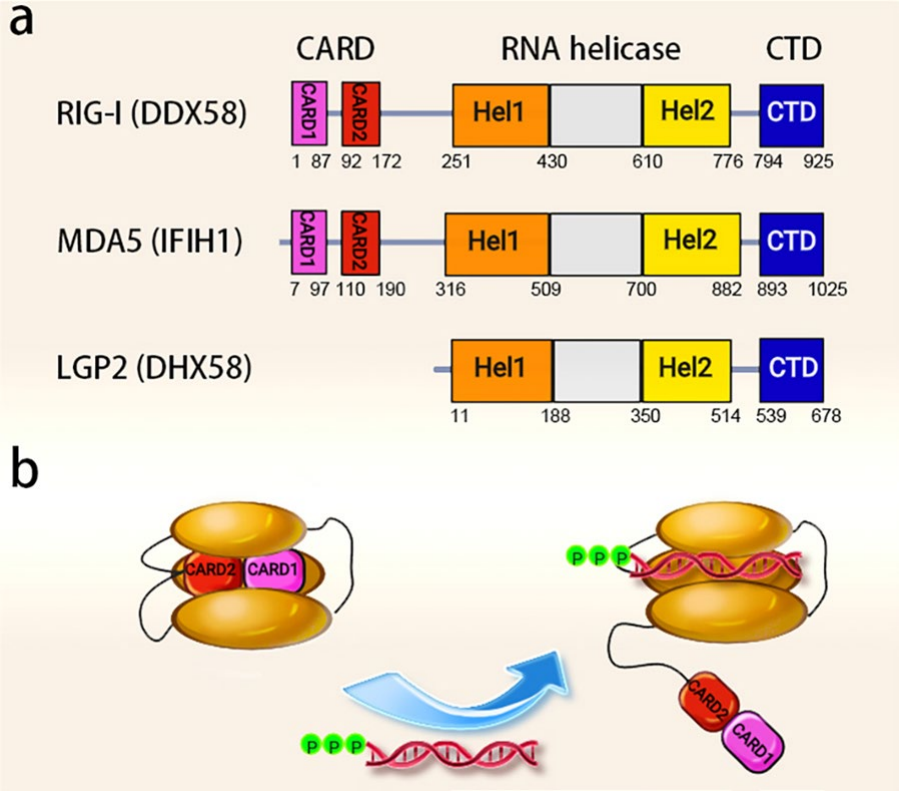
Schematic illustration of the domains of RLRs. **a** Both RIG-I and MDA5 have two N-terminal tandem caspase activation and recruitment domains (CARDs), two central Rec A domains (Hel‐1 and Hel‐2), and a C-terminal domain (CTD), while LGP2 is lack of CARD. **b** Under resting states, the CARDs of RIG-I are masked by an autoinhibitory conformation. The binding of 5′-ppp dsRNA to RIG-I triggers the unmasking of CARDs that allows signal transmission

RIG-I-like receptors (RLRs) are intracellular PRRs that detect pathogenic RNA species generated during infection by RNA viruses, DNA viruses and some bacteria. The RLRs family comprises retinoic acid-inducible gene 1 (RIG-I, also called DDX58), melanoma differentiation-associated gene 5 (MDA5, also called IFIH1) and laboratory of genetics and physiology 2 (LGP2, also called DHX58). Activation of RLRs leads to the transcriptional induction of type I/III interferons and other cytokines that reinforce the immune responses and induce the expression of proteins capable of interfering with the life cycle of pathogens [19]. RIG-I and MDA5 share similar structure and function but differ in the preference for RNA ligands. While RIG-I preferentially binds to short double-stranded RNAs (dsRNAs), MDA5 detects long accessible dsRNAs or RNA aggregates [20, 21]. Both RIG-I and MDA5 contain two N-terminal tandem caspase activation and recruitment domains (CARDs) that are required for signal transmission, two central Rec A domains (Hel‐1 and Hel‐2) that have DExH‐box‐type RNA helicase activity, and a C-terminal domain (CTD) that collaborates with the helicase domain to detect immunostimulatory RNAs (Fig. 1a) [22, 23]. In uninfected cells, the CARDs of RIG-I and MDA5 are masked by an autoinhibitory conformation that prevents downstream signal transduction [24]. Following viral infection, viral RNAs bind both the CTD and the helicase domain in RLRs and activate the ATPase activity, leading to a conformational change and unmasking of CARDs (Fig. 1b) [23, 25]. The CARDs of RIG‐I and MDA5 then interact with mitochondrial antiviral signaling protein (MAVS) that triggers antiviral interferon (IFN) responses [23, 25].

Interferons are important components of the host innate immune response. Type I IFNs (IFN-α, -β, -ε and -Ω) engage IFN-α/β receptor (IFNAR) and activate the Janus kinase (JAK)–signal transducer and activator of transcription (STAT) pathway, leading to the expression of interferon-stimulated genes (ISGs). RLRs-mediated expression of IFNs and ISGs may have both beneficial and detrimental effects on the host. RIG-I-deficient mice are susceptible to both virus- and bacteria-induced inflammation [26]. However, viruses can escape from RLRs-mediated immune surveillance by multiple mechanisms. Given that RLRs play critical roles in triggering host immune response and suppressing inflammation-associated carcinogenesis, they are promising targets for cancer immunotherapy. In this review, we introduce the latest advances in exploiting RIG-I and MDA5 for cancer immunotherapy. The RIG-I/MDA5-targeted therapy can be integrated with other cancer immunotherapies such as CAR-T cells and immune checkpoint blockade [27, 28].

## An overview of RLRs signaling

**Fig. 2:**
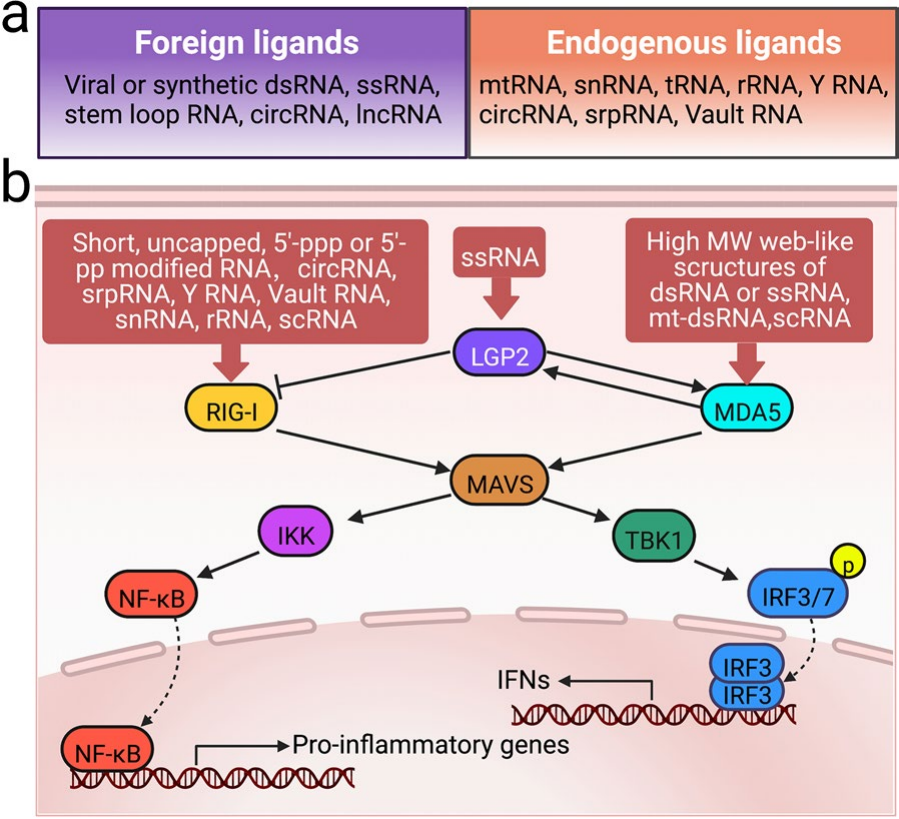
Ligands and signaling pathways of RLRs. **a** RLRs may be engaged by both foreign (non-self) RNA and endogenous (self) RNA. **b** RLRs have a preference for different RNA. Stimulation of RLRs by RNA ligands leads to MAVS-mediated activation of TBK1-IRF3/7 and IKK-NF-kB signaling pathways, which in turn induce the expression of IFNs and proinflammatory genes. circRNA, circular RNA. srpRNA, signal recognition particle RNA. dsRNA, double-stranded RNA. lncRNA, long noncoding RNA. mt-dsRNA, mitochondrial dsRNA. rRNA, ribosome RNA. ssRNA, single-stranded RNA. tRNA, transfer RNA. MW, molecular weight

The detection of RNA and initiation of innate immune response by RLRs is a mechanism of combating viral or bacterial infection (Fig. 2). The classical RIG-I ligand is uncapped 5'-tri- or 5'-di-phosphate RNA (5'-pppRNA or 5'-ppRNA), which can also be generated by RNA polymerase III (RNAPOLIII) after binding to AT-rich double-stranded DNA (dsDNA) [29, 30]. Thus, RIG-I may be activated by both RNA and DNA viruses [31]. Of note, RIG-I can also be activated by foreign circRNA (circular RNA) independent of a 5' triphosphate and dsRNA structure [32]. Discrimination between viral and cellular (self) RNA is crucial in maintaining effective antiviral interferon response while avoiding autoimmunity. Uncapped 5'-pppRNA are generated during viral replication, whereas self-RNAs generated during normal cellular metabolism are 5'-end capped or monophosphorylated [33]. Moreover, N6-methyladenosine (m6A) modification of RNA may be another mechanism for the host to discriminate self-RNA from non-self-RNA [32]. Viral RNA with m6A modification poorly binds to RIG-I, whereas m6A-deficient virion RNA binds more efficiently to RIG-I and potently induces interferon expression [34, 35]. m6A modification is also attributable to the discrimination between foreign and endogenous circRNA [36]. In addition, A to I editing of endogenous dsRNA by adenosine deaminase acting on RNA 1 (ADAR1) can prevent sensing of self-RNA by MDA5 and triggering MAVS-mediated type I interferon response [35, 37, 38]. Nevertheless, RLRs can sense endogenous RNA and even DNA that are mislocalized or misprocessed in cells [33, 39–42]. The endogenous noncoding RNAs associated with RIG-I include small nucleolar RNA (snRNA), signal recognition particle RNA (srpRNA), transfer RNA (tRNA), vault RNA, Y RNA and retrotransposon-derived RNAs [33, 40, 41]. In addition, the accumulation of mitochondrial dsRNA species in the cytoplasm may induce interferon response through the MDA5–MAVS axis, especially when the mitochondrial dsRNA is cleaved by RNAase L [42, 43].

While LGP2, RIG-I and MAD5 have similar RNA-binding capacity, there are no N-terminal CARDs in LGP2 that are necessary for signaling transmission. Hence, LGP2 usually acts as a regulator of RIG-I and MDA5 [44]. LGP2 may repress RIG-I signaling through multiple mechanisms, such as interruption of the interaction between RIG-I and MAVS [45, 46], inhibition of Dicer processing of long dsRNA [47], prevention of viral dsRNA binding to RIG-I [48], and suppression of TRIM25-mediated RIG-I ubiquitination [49]. On the other hand, accumulating evidence indicates that LGP2 cooperates with MDA5 to augment IFN response [50–52]. LGP2 promotes MDA5 nucleation and the conversion of MDA5 to an active conformation [50]. Therefore, LGP2 can be both RLR coactivator and corepressor depending on the context. LGP2 may act as an off-switch regulator of RIG-I and an on-switch regulator of MDA5. Nevertheless, LGP2 is not required for the IFN responses to synthetic RNA ligands for MDA5 and RIG-I [53].

Once RIG-I and MDA5 are activated by RNA, the exposed RIG-I/MDA5 CARDs interact with the mono CARD domain of MAVS, an outer mitochondrial membrane protein, and induce MAVS oligomerization [54]. Oligomeric MAVS further initiates TRAF2/3/5/6 activation, which sensitizes TBK1 to activate several transcription factors including IRF3/7 and then induces the production of IFN and cytokines [55, 56]. In addition, the IkappaB kinase complex (IKK) is activated by MAVS, which in turn activates nuclear factor-kappa B (NF-kB) and induces the expression of proinflammatory genes (Fig. 2).

## Regulation of RLRs by ubiquitination and de-ubiquitination

Both RIG-I and MDA5 are subject to posttranslational modifications. Ubiquitination or polyubiquitin binding is essential for the activation of RIG-I and MDA5 [57–59]. The E3 ubiquitin ligase TRIM25 (tripartite motif protein 25) delivers the lysine 63 (K63)-linked polyubiquitin moiety to the CARD domains of RIG-I and MDA5, leading to efficient interaction between RIG-I/MDA5 and MAVS [60, 61]. On the other hand, the ubiquitin-specific protease USP15 promotes RIG-I-mediated antiviral immunity by deubiquitylating and stabilizing TRIM25 [60]. Nuclear Dbf2-related kinase 2 (NDR2) and ERA G-protein-like 1 (ERAL1) directly interact with RIG-I and TRIM25, thereby promoting TRIM25-mediated K63-linked polyubiquitination of RIG-I and antiviral immune response [62, 63]. In addition, K63-linked polyubiquitination of the CTD domain in RIG-I and MDA5 promotes their activation. Riplet (also called Reul or RNF135) and TRIM65 mediate K63-linked polyubiquitination of RIG-I and MDA5 CTDs, respectively [64–67]. In contrast, the deubiquitinase OTUD3 binds to RIG-I/MDA5 and removes K63-linked ubiquitination, leading to reduced binding of RIG-I and MDA5 to viral RNA and the downstream adaptor MAVS [68]. Also, the deubiquitinases CYLD, USP3, USP14 and USP27X physically interact with RIG-I and cleave the K63-linked polyubiquitin chains, thereby attenuating the antiviral immunity [69–72]. To restore immune homeostasis and prevent excessive inflammation, the endoplasmic reticulum-resident protein reticulon 3 interacts with both TRIM25 and RIG-I, thereby impairing the ubiquitination of RIG-I by TRIM25 and inhibiting both IRF3 and NF-κB activation [73].

Unlike Riplet and TRIM25, the ubiquitin ligases RNF125, TRIM40 and Parkin mediate K48- or K27-, but not K63-linked polyubiquitination of RIG-I and MDA5, leading to proteasomal degradation of RIG-I and MDA5 [74–76]. RIO kinase 3 (RIOK3) facilitates the interaction between TRIM40 and RIG-I/MDA5, thereby enhancing RIG-I/MDA5 degradation [77]. In fact, there are many proteins that contribute to the degradation of RIG-I and MDA5 by the proteasome. RLRs signaling usually occurs at the endoplasmic reticulum–mitochondrial contact sites. The endoplasmic reticulum-resident p97 complex directly binds both RNF125 and non-ubiquitinated RIG-I and then promotes K48-linked ubiquitination of RIG-I at residue K181 [78]. RNF122 also delivers the K48-linked ubiquitin to the K115 and K146 residues of RIG-I CARDs and promotes RIG-I degradation [79]. In addition, the E3 ubiquitin ligase CHIP/STUB1 promotes K48-linked polyubiquitination and proteasomal degradation of RIG-I, which is facilitated by cytoplasmic MLL5 through increasing RIG-I and STUB1 association [80]. The ubiquitin ligase MEX3A interacts with RIG-I and induces its ubiquitylation and proteasomal degradation [81], whereas MEX3C promotes K63-linked ubiquitination of RIG-I and stimulates IFN production [82]. The ubiquitin ligases may also have opposing roles in regulating RIG-I and MDA5. For example, TRIM13 negatively regulates MDA5-mediated type I IFN production but positively regulates RIG-I signaling [83]. Except for ubiquitin, the ubiquitin-like protein FAT10 is recruited to RIG-I by ZNF598, resulting in the inhibition of RIG-I polyubiquitination and IFN response [84].

## Regulation of RLRs by phosphorylation and SUMOylation

Phosphorylation of RIG-I protein is a mechanism underlying the prevention of RIG-I activation under normal conditions. Protein kinase C-α (PKC-α) and PKC-β are the primary kinases responsible for RIG-I S8 and T170 phosphorylation, which prevent TRIM25 binding and TRIM25-mediated polyubiquitination of RIG-I [85]. In addition, casein kinase II (CK2) phosphorylates T770 and S854/855 residues in the C-terminal domain of RIG-I and thereby silences RIG-I signaling at resting state [86]. Furthermore, RIG-I phosphorylation is a mechanism of fine-tuning RIG-I activity and preventing immunopathology. Death-associated protein kinase 1 is activated by RIG-I and reciprocally phosphorylates T667 residue in RIG-I to inhibit dsRNA binding [87]. Phosphorylation of MDA5 at S88 and S828 also keeps it in an inactive state [88, 89]. RIOK3 is responsible for phosphorylating S828 residue in the C-terminal domain of MDA5 [85]. Phosphorylation of MDA5 S828 impairs MDA5 oligomerization and suppresses its signaling [89]. In contrast, dephosphorylation of RIG-I and MDA5 by protein phosphatase 1 (PP1) is essential for the activation of RIG-I and MDA5 [88]. Following viral infection or nanoparticle exposure, the actin cytoskeleton is remodeled, allowing the PP1 regulatory subunit PP1R12C to dissociate from filamentous actin and interact with cytoplasmic RLRs, thereby dephosphorylating RIG-I and MDA5 [90]. Dephosphorylation primes RLRs for RNA binding and subsequent activation.

In addition, RIG-I and MDA5 are regulated by SUMOylation. SUMOylation of the CARD domains of RIG-I and MDA5 by TRIM38 prevents their K48-linked polyubiquitination and degradation, and their dephosphorylation by PP1 following viral infection [91]. The SUMOylation E3 ligase PIAS2β and SUMO-conjugating enzyme Ubc9 also induce MDA5 SUMOylation and activation, but do not affect K48-linked polyubiquitination and degradation [92]. It remains unclear whether PIAS2β SUMOylates the C-terminal domain of MDA5, and whether PIAS2β promotes MDA5 oligomerization. In contrast, both RIG-I and MDA5 are deSUMOylated by SENP2, which promotes K48-linked polyubiquitination and degradation [91]. Lastly, RIG-I is subject to acetylation. Acetylation of K909 in the CTD of RIG-I prevents dsRNA binding to RIG-I [93]. Deacetylation of RIG-I by HDAC6 primes RIG-I activation upon viral infection [89]. It is unclear whether MDA5 is also subject to regulation by acetylation.

## Rationale for therapeutic targeting RLRs in cancer

Both viral and bacterial infections may induce carcinogenesis. The DNA virus HBV and the RNA virus HCV can be detected by cytosolic RLRs in host cells [94–98]. In addition, EBV may be sensed by RIG-I via EBV-encoded small RNAs (EBER1/2) that are transcribed by RNAPOLIII [99]. Upon H. pylori infection, gastric epithelial cells also produce type I IFNs and ISGs in RIG-I-dependent manner [100]. RIG-I can be activated by 5′-pppRNA from intracellular H. pylori [100]. While host innate immunity is critical for the suppression of viral and bacterial infection, pathogens can evade host immune responses through complex mechanisms. Both HBV and HCV transcripts are subject to m6A modification, which is an important RNA modification to regulate RNA stability and translation [101]. m6A modification of HBV/HCV transcripts prevents the recognition of viral RNAs by RIG-I, thereby promoting immune evasion [101]. The mechanisms by which EBV suppresses RLRs-mediated innate immunity are more complex. The EBV immediate-early protein BRLF1 interacts with RNAPOLIII to inhibit EBER transcription, thereby suppressing RIG-I activation and antiviral responses [102]. In addition, the EBV large tegument protein BPLF1 sequesters TRIM25 and prevents the ubiquitination of RIG-I by TRIM25, leading to impaired RIG-I signaling [103]. Meanwhile, EBV-encoded LMP1 can promote proteasomal degradation of RIG-I by recruiting the E3 ubiquitin ligase carboxyl-terminus of Hsp70 interacting protein (CHIP) to RIG-I [104]. EBV miR-BART6-3 ps also targets the 3'UTR of RIG-I mRNA and inhibits RIG-I expression [105]. Evasion of RIG-I-mediated innate immune responses may help EBV-infected cells transformation. Except for viruses, H. pylori also actively suppresses STING and RIG-I signaling via the downregulation of IRF3 activation [106]. Decreased RIG-I expression is associated with poor prognosis and promotes cell invasion in human gastric cancer and HCC [107, 108].

The above-described evidence demonstrates that pathogens have evolved mechanisms allowing them to evade host immunity, damage the target tissues and promote carcinogenesis. Except for pathogen-derived effectors, cancer cells can also disable RLRs signaling through intrinsic factors. Epigenetic repression of RIG-I transcription may contribute to reduced expression of RIG-I in cancer. Decreased levels of H3K4me3 but increased H3K9me3 and H3K27me3 in HCC may lead to reduced expression of RIG-I in HCC [108]. In addition, overexpression of MEX3A, a protein that promotes RIG-I degradation, is detected in some types of cancer [81]. Downregulation of RLRs not only suppresses innate immunity but also dampens the subsequent adaptive immune responses. The compromised host immune defense further promotes cancer progression, even after pathogens are cleared.

**Fig. 3:**
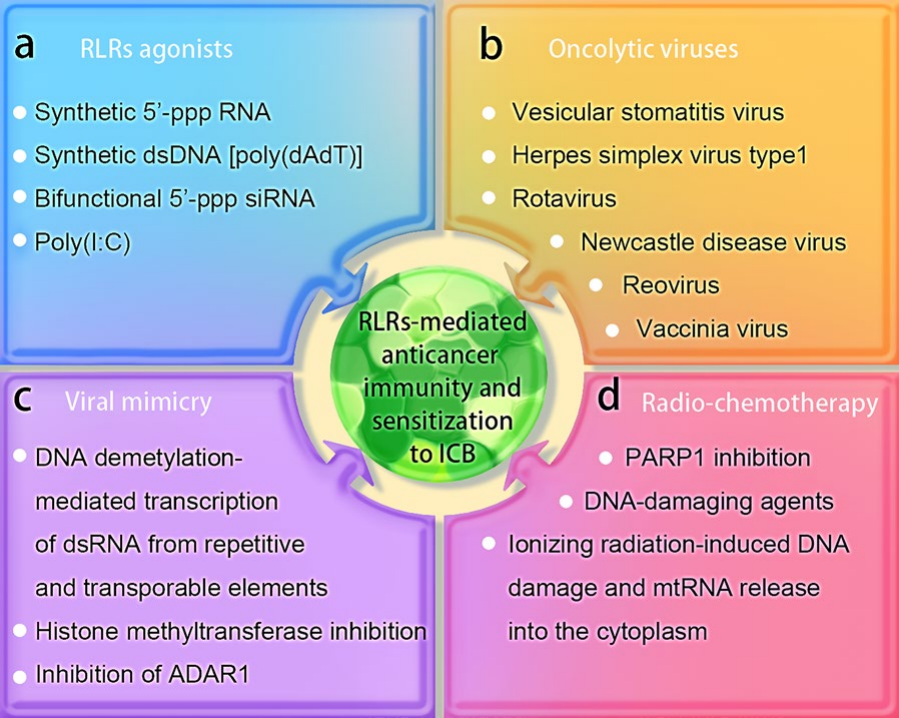
Therapeutic targeting RLRs in cancer. **a** RLRs can be activated by synthetic RNA, dsDNA or bifunctional 5′-ppp siRNA. **b** Oncolytic virus infection may induce RLRs signaling and sensitize tumors to immune checkpoint blockade (ICB). **c** Viral mimicry is a strategy to activate RLRs-mediated antitumor immunity. **d** Radio-chemotherapy-induced DNA damage and mitochondrial RNA (mtRNA) release trigger RLRs signaling and sensitize tumors to ICB

Accumulating evidence suggests that the presence of intact type I IFN signaling is critical for the efficacy of many conventional chemotherapeutics and targeted anticancer agents [109]. Given that RLRs are critical for activating the IFN responses and inducing immunogenic cell death, stimulation of RIG-I or MDA5 signaling has emerged as a strategy for cancer therapy. For infection-associated cancer, the intactness of core elements in RIG-I and MDA5 signaling pathways may be taken into account to determine whether RIG-I or LGP2/MDA5 agonists are appropriate for the induction of anticancer immunity. Cancer immunotherapy, such as immune checkpoint blockade, has achieved remarkable success in the treatment of cancer. However, non-inflamed (“immune-cold”) tumor is not sensitive to immune checkpoint inhibitors (ICI). Stimulation of RLRs signaling may increase the proinflammatory phenotype and prime the tumor microenvironment for ICI response [110]. In addition, RIG-I and MDA5 can induce type I IFN-independent apoptosis in some types of cancer [111]. Therefore, stimulation of RLRs signaling may facilitate the immunotherapy of “immune- cold” tumors regardless of the etiology. The RLRs signaling can be induced by synthetic RNA oligonucleotides, oncolytic viruses, viral mimicry and radio-chemotherapy (Fig. 3).

## Treatment of cancer with synthetic 5′-pppRNA

5′-pppRNA, 5′-ppp stem-loop RNA (5′ppp-SLR) and 5′-ppRNA sequences can act as powerful RIG-I agonists [112]. While the double-strand polyriboinosinic-polyribocytidylic acid (poly I:C) is a MDA5 and TLR3 ligand bearing either monophosphate or diphosphate, shortening poly(I:C) converts it into a RIG-I ligand [113]. In addition, polydeoxyadenosine-deoxythymidine (poly dAdT), a synthetic dsDNA, also indirectly stimulates RIG-I through an RNA polymerase III–mediated transcription into 5′-pppRNA [114, 115]. Preclinical studies have demonstrated the efficacy of RIG-I agonists in several cancer types [116–118]. The sequences of some RNA agonists of RIG-I are given in Table 1 [117, 119–127]. While RIG-I agonists may directly induce cancer cell death in vitro [125], intratumoral-delivered RIG-I agonist SLR14 is mainly taken up by CD11b^+^ tumor-infiltrating myeloid cells, which further increases the tumor-infiltrating CD8^+^ T cells, NK cells, and CD11b^+^ myeloid cells while reducing the immunosuppressive CD4^+^FoxP3^+^ T reg cells [119]. Treatment with RIG-I agonists may also promote antigen presentation, dendritic phagocytosis, and the expression of proinflammatory cytokines [125]. The tumor-suppressing effects of RIG-I activation involve the crosstalk among cancer cells, immune cells and endothelial cells. Activation of RIG-I in natural killer (NK) cells leads to the secretion of TRAIL, which induces cancer cell death [118]. On the other hand, stimulation of RIG-I in melanoma cells induces the secretion of extracellular vesicles harboring the NKp30-ligand (BAG6, BAT3) on their surface, which engages NK cell receptor NKp30 and thereby induces NK cell-mediated lysis of melanoma cells [128]. In addition, viral infection and dsRNA or dsDNA may increase RIG-I expression in endothelial cells [129–131]. Stimulation of RIG-I in endothelial cells induces vascular oxidative stress [132].

**Table 1:** Selective RIG-I agonists and their effects on tumorigenesis

Name	Property	Target	Sequences	Effects on tumorigenesis	References
/	Hairpin RNA	RIG-I	5′-pppGCGCUAUCCAGCUUACGUAGAG CUCUACGUAAGCUG GAUAGCGC-3′	Activation of RIG-I in melanoma cells in vitro	[117]
SLR14	Stem-loop RNA	RIG-I	5′-pppGGAUCGAUCGUUCGCGAUCGAU CGAUCC-3′	Potent antitumor effect in immunogenic or poorly immunogenic melanoma	[119]
/	ssRNA	RIG-I	5′-pppGGGGCUGACCCUGAAGUUCAUC UU-3′	Not determined	[120]
/	ssRNA	RIG-I	5′-pppGGGGAUGAACUUCAGGGUCAGC UU-3′	Not determined	[120]
Poly-U/UC	ssRNA	RIG-I	5′-pppGGCCAUCCUG(U7)CCC(U11)C (U34)CUCC(U9)CCUC(U7)CC(U4)CUUUCCUUU-3′	Not determined	[121]
/	dsRNA	RIG-I	Sense: 5′-pppGCGCUAUCCAGCUUACGU AG-3′ Antisense: 5′-pppCUACGUAAGCUGGAU AGCGC-3′	Significant local and systemic antitumor effects and survival benefits in murine B16-F10 melanoma model; sensitization of AML to anti-PD1 antibody	[117] [122]
RN7SL1	ncRNA	RIG-I	5′-GCCGGGCGCGGUGGCGCGUGCCUGU AGUCCCAGCUACUCGGGAGGCUGAGGCUGGAGGAUCGCUUGAGUCCAGGAGUUCUGGGCUGUAGUGCGCUAUGCCGAUCGGGUGUCCGCACUAAGUUCGGCAUCAAUAUGGUGACCUCCCGGGAGCGGGGGACCACCAGGUUGCCUAAGGAGGGGUGAACCGGCCCAGGUCGGAAACGGAGCAGGUCAAAACUCCCGUGCUGAUCAGUAGUGGGAUCGCGCCUGUGAAUAGCCACUGCACUCCAGCCUGGGCAACAUAGCGAGACCCCGUCUCU-3′	Activation of RIG-I in breast cancer cells by RN7SL1 promotes tumor growth and metastasis; delivery of RN7SL1 by CAR-T cells inhibits B16 melanoma growth when combined with peptide vaccine or immune checkpoint blockade	[27] [123] [124]
M8	dsRNA	RIG-I	Sense: 5′-pppGAAAUUAAUACGACUCAC UAUAGACGAAGACCACAAAACCAGAU(A26)UAA(U26)AUCUGGUUUUGUGGUCUUCGUC-3′ Antisense: 5′-pppGACGAAGACCACAAAA CCAGAU(A26)UUA(U26)AUCUGGUUUUGUGGUCUUCGUCUAUAGUGAGUCGUAUUAAUUUC-3′	Induction of IFN-I-dependent melanoma cell death and stimulation of the phagocytic potential of dendritic cells	[125]
/	dsRNA	RIG-I	Sense: 5′-pppUCAAACAGUCCUCGCAUG CCUAUAGUGAGUCG-3′ Antisense: 5′-pppGCAUGCGAGGACUGUU UGACUAUAGUGAGUCG-3′	Complete regression of pre-established B16 melanoma when combined with ovalbumin vaccine and anti-CTLA4 antibody	[126]
3p-siBCL2	Bifunctional siRNA	MurineBCL2 RIG-I	Sense: 5′-pppUCAAACAGAGGUCGCAUG CCUAUAGUGAGUCG-3′ Antisense: 5′-pppGCAUGCGACCUCUGUU UGACUAUAGUGAGUCG-3′	Significant antitumor efficacy in melanoma and colon carcinoma models;	[127]

Of note, the sensitivity of RIG-I agonists may be regulated by host factors. This may be taken into account when RIG-I agonists are used to treat cancer. PTPN11 is an RNA phosphatase that can dephosphorylate 5′-pppRNA [133]. Hence, inhibition of PTPN11 may enhance the stability of 5′-pppRNA. Nudix Hydrolase 2 (NUDT2) is another protein that can remove 5′-phosphorylates from RNA and then destabilize RNA [134]. Inhibition of NUDT2 may also improve the efficacy of 5′-pppRNA. Moreover, a previous study has identified lactate as a natural suppressor of RLR signaling [135]. Aerobic glycolysis, a hallmark of cancer, promotes lactate production in tumors. It remains to know whether glycolytic metabolism and its targeting may affect the responsiveness of RIG-I to its agonists.

## Treatment of cancer with bifunctional 5′-ppp siRNA

To enhance the efficiency of 5′-pppRNA, bifunctional 5′-ppp siRNA has been developed to simultaneously activate RIG-I-mediated immune responses and suppress the expression of oncogenes or drug resistance genes. Multidrug Resistance Protein 1 (MDR1), a member of the superfamily of ATP-binding cassette transporters, is an ATP-dependent drug efflux pump for xenobiotic compounds with broad substrates. It reduces drug accumulation in multidrug-resistant cells and often mediates the development of resistance to anticancer drugs. One study shows that the treatment of leukemia cells with 5′-ppp siRNA targeting MDR1 inhibits MDR1 expression and drug resistance, and activates RIG-I signaling [136]. However, this study did not demonstrate whether 5′-ppp siMDR1 has superior anticancer effects to siMDR1 or 5′-pppRNA in vivo. In addition, BCL2 is an antiapoptotic protein that promotes tumor cell survival and drug resistance. Compared with siBCL2 and 5′-pppRNA, 5′-ppp siRNA targeting BCL2 more profoundly suppresses melanoma growth and metastasis in murine models of melanoma [127]. RIG-I-dependent type I IFN induction in both tumor cells and CD11c^+^ dendritic cells has a critical role in mediating the anticancer effects of 5′-ppp siBCL2 [127]. Meanwhile, the anticancer activity of 5′-ppp siBCL2 in the B16 melanoma model depends on NK cells but not CD8^+^ T cells [127] which may be attributable to type I IFN-induced change in major histocompatibility complex (MHC) molecules and other ligands on B16 melanoma cell surface, allowing the recognition of these tumor cells specifically by NK cells [137]. Nevertheless, it does not exclude the possibility that CD8^+^ T cells may contribute to the anticancer effects of 5′-ppp siBCL2 in other contexts. Another study shows that the 5′-ppp siRNA targeting VEGF inhibits tumor angiogenesis and induces innate immune responses and massive tumor necrosis in a murine model of lung cancer [138]. In an orthotopic mouse model of pancreatic cancer, the bifunctional 5′-ppp siRNA targeting TGF-β exhibits superior anticancer effects compared with 5′-pppRNA or TGF-β siRNA, which is largely dependent on the recruitment of activated CD8^+^ T cells to the tumor [139]. Given that TGF-β is an immune-suppressive and pro-metastasis factor [140–142], this bifunctional TGF-β siRNA may stimulate RIG-I-mediated immune responses, break TGF-β-mediated immune evasion and suppress cancer metastasis. In addition, the 5′-ppp siRNA targeting glutaminase, a key enzyme in glutamine metabolism, not only induces RIG-I-mediated reactive oxygen species generation and immune responses but also impairs glutaminase-mediated ROS scavenging, thereby triggering prominent tumor cell apoptosis [143].

## In vivo delivery of RLR-activating RNA by nanoparticles, extracellular vesicles and CAR-T cells

**Fig. 4:**
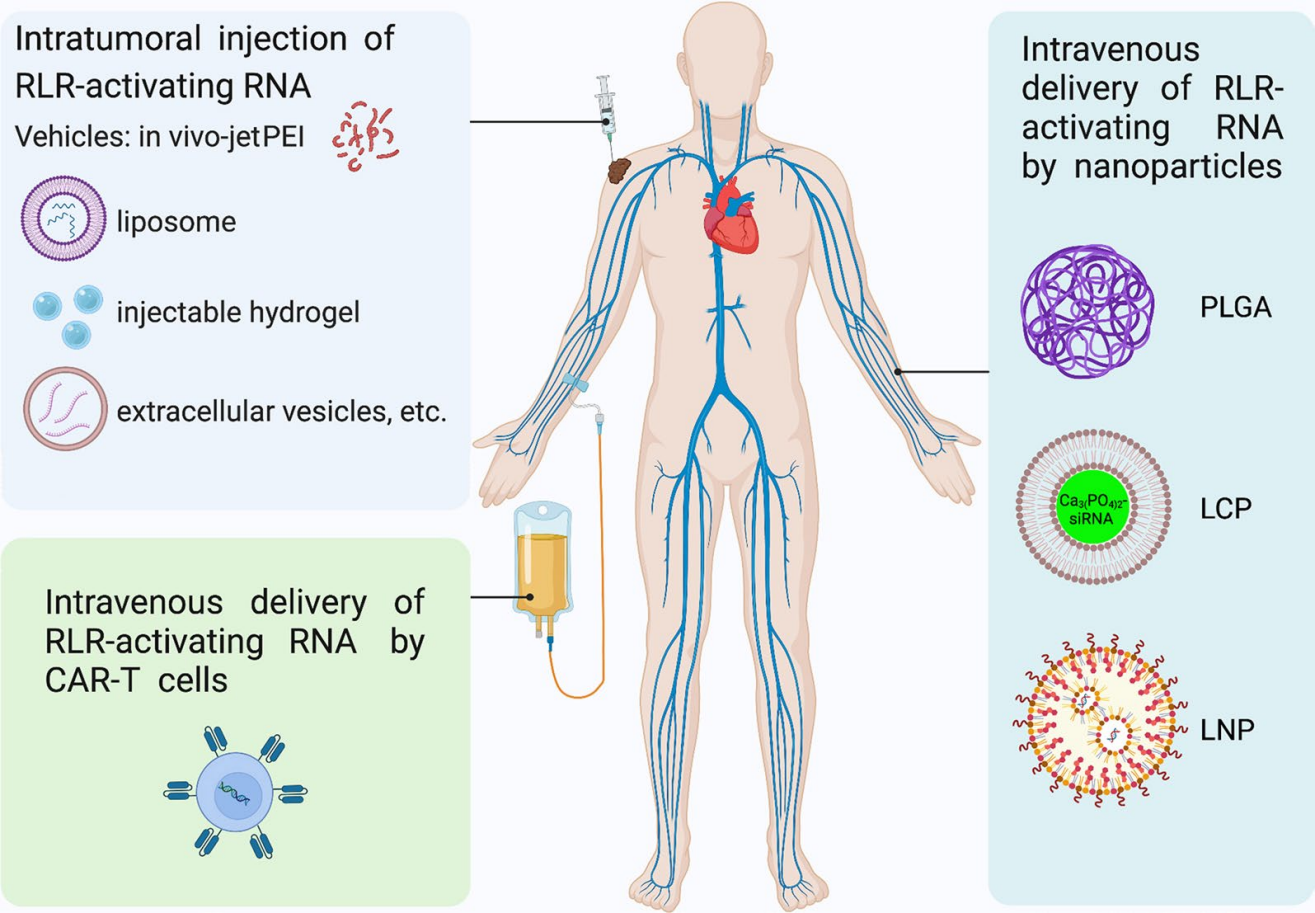
Intratumoral and intravenous delivery of RLR-activating RNA. In vivo jetPEI, liposome, injectable hydrogel and extracellular vesicles can serve as the vehicles for intratumoral delivery of RLR-activating RNA. Systemic delivery of RLR-activating RNA can be achieved by intravenous injection of nanoparticles such as carboxylic acid-terminated poly(lactic-*co*-glycolic acid) (PLGA), lipid–calcium–phosphate (LCP) and lipid nanoparticles (LNP). In addition, CAR-T cells can deliver and release the RLR-activating RNA

While both 5′-pppRNA and 5′-ppp siRNA are promising anticancer agents, in vivo delivery of these RNA oligonucleotides is still challenging. Recently, technological advances in RNA delivery systems have been achieved to improve the safety and activity of small RNA therapeutics. As a negatively charged hydrophilic molecule, RNA needs some formulations for cell entry. Liposome, which was first described by Alec Bangham in 1960s, is a widely used reagent to deliver DNA and RNA into the cells [144]. Liposomal vesicles are composed of phospholipids or synthetic amphiphiles incorporated with cholesterol [145]. Intratumoral liposome delivery of poly(I:C) induces RIG-I/MDA5 expression and inhibits the growth of hepatoma and gastric cancer xenografts [146, 147]. Also, microparticles, nanoparticles and hydrogels are representative carriers of siRNA, miRNA and 5′-pppRNA [148] (Fig. 4). Intratumoral delivery of 5′-ppp RNA by a pH-responsive, membrane-destabilizing dimethylaminoethyl methacrylate-*b*-(dimethylaminoethyl methacrylate-*c*-butyl methacrylate-*c*-propylacrylic acid) nanoparticles can resist endosomal/lysosomal degradation of RNA and potently activate RIG-I [149]. In preclinical studies, systemic delivery of nanoparticles composed of carboxylic acid-terminated poly(lactic-*co*-glycolic acid) (PLGA), 5′-ppp dsRNA and other innate agonists induces anticancer effects in murine melanoma model [150]. Compared with intratumoral delivery, systemic delivery of RIG-I agonists may be more feasible and effective in many clinical settings.

The systemic delivery of RIG-I-activating RNA may also be achieved by the lipid–calcium–phosphate (LCP) nanoparticle platform (Fig. 4). This platform integrates both cationic lipid–protamine–nucleic acids complexes and calcium phosphate precipitates [151]. For the preparation of cationic lipid–protamine–nucleic acids complexes, DNA/RNA first interacts with protamine sulfate, a cationic polypeptide, and then incubates with DOTAP (1,2-dioleoyl-3-trimethylammonium-propane) cationic liposomes, producing the positively charged nanoparticles that are further modified by double-chain phospholipid conjugate of polyethylene glycol (PEG) tethered with anisamide [152]. While the cationic lipid-protamine-RNA complexes can remain stable in circulation after intravenous injection and successfully deliver RNA, the release of RNA into the cytoplasm is inconsistent among different cells [153]. This problem is overcome by the successful development of LCP nanoparticles by replacing the core of cationic lipid–protamine–nucleic acids complexes with nanosized calcium phosphate precipitates in which RNA is entrapped [154]. Intravenous injection of LCP nanoparticles encapsulating a bifunctional 5′-ppp siRNA also has antitumor efficacy in murine models of pancreatic adenocarcinoma, while it does not induce systemic immunomodulation [155].

While the traditional liposome can associate with negatively charged DNA and RNA to build a hydrophobic nanoparticles system, the usefulness of this system in vivo is limited by the toxicity of positively charged lipids. Dr. Villus’s group developed the ionizable lipid nanoparticles (LNPs) system that is lack of massive toxicity in vivo [156]. These types of LNPs are cocktails of amphipathic phospholipid, ionizable amino lipid, polyethylene glycol (PEG) lipid and cholesterol [156, 157]. The ionizable amino lipid directly binds to nucleic acids and facilitates endosomal escape. Amphipathic phospholipid helps with the fusion of LNP with cell and endosomal membranes [158]. The stability of LNP is enhanced by cholesterol [159]. One of the biggest breakthroughs in this field is the liver-targeted delivery of siRNA by LNP [156, 160]. The first liver-targeted siRNA-LNP drug, Onpattro® (Patisiran) has been approved by US FDA and European Medicines Agency [161]. Later on, the LNP system was used to deliver mRNA in vivo. Drs. Weissman and Karikó then took advantage of the LNP system to develop mRNA vaccines. Impressively, the LNP technology enables the rapid development of mRNA vaccines by BioNTech/Pfizer and Moderna to fight against COVID-19 [145]. Microfluidic mixing is a general technique for formulating an LNP-RNA delivery system [162]. So far, there are little reports on the in vivo delivery of RLR-activating RNA to treat cancer. It warrants more studies to exploit this advanced delivery system for treating cancer by RLR-activating RNA.

When the RIG-I agonists are systemically delivered, tumor tissue targeting and off-target toxicities are critical concerns. While the traditional LNP usually delivers its payload to the liver, the LNP formulations can potentially be redesigned to deliver RNA agonists of RLR to other sites for treating different cancers. The liver-, lung- and spleen-specific delivery of RNA has been achieved by selective organ-targeting nanoparticles in which an organ-specific targeting molecule is included in the basic four-component LNPs [163]. The liver- or lung-specific delivery of RLR-activating RNA may also be achieved by the organ-specific LNPs. In addition, recent studies have demonstrated that modifying the ionizable lipid in the traditional LNPs can deliver RNA to immune cells [164]. Adding phosphatidylserine into the standard four-component LNPs also efficiently delivers RNA to lymph nodes after IV administration [165]. These emerging platforms may stimulate more studies to determine how immune cells-specific delivery of RLR-activating RNA may affect tumor progression. Except for liver-, lung- and lymph node-specific delivery of RNA, the specific delivery of RNA to other organs after intravenous administration may enhance the utility of LNPs. In addition, extracellular vesicles (EVs) are an emerging platform for delivering siRNA, peptides or proteins [166, 167]. EVs from red blood cells have been successfully used for intratumoral delivery of 5'-pppRNA as RIG-I agonist [168]. It warrants further studies to determine whether engineering EVs can allow intravenous delivery of RIG-I agonists to tumors.

Engineering chimeric antigen receptor (CAR)-T cells with tumor specificity have made impressive success in the treatment of patients with hematologic malignancies [169–174]. However, the efficacy of CAR-T therapy in many solid tumors remains poor. CAR-T cells have been engineered to produce the unshielded noncoding RNA RN7SL1 and release it via EVs [27]. While RN7SL1 activates RLR signaling in CAR-T cells and improves CAR-T cell function, the RN7SL1-loaded EVs are preferentially transferred to immune rather than tumor cells in the tumor microenvironment [27]. The mechanism underlying the selective delivery of the RN7SL1-loaded EVs is elusive. Stimulation of RLRs in immune cells by RN7SL1 enhances anticancer immunity and enables tumor suppression when immune checkpoints are blocked [27]. Taking advantage of CAR-T cells to deliver RLRs agonists into the tumor microenvironment is a promising strategy for cancer immunotherapy.

## Stimulation of RLR signaling for cancer immunotherapy by oncolytic virus

RLRs are major sensors of RNA virus infection, indicating that activation of RLRs for cancer therapy may be achieved by virus infection. Indeed, RLRs signaling is involved in the antitumor effects of some oncolytic viruses [175]. Oncolytic RNA or DNA viruses are replication-competent viruses that can infect and lyse cancer cells. While some native viral species are capable of inducing immunogenic cell death in tumor cells, genetic engineering by introducing transgenes or modifying viral genes can enhance their tumor selectivity and the competence of replication and antitumor immunity [175]. Also, oncolytic viruses have been used to modulate the tumor microenvironment and complement conventional treatments or other immunotherapies [175–177]. Importantly, virus replication is not required for the antitumor immunity of some types of oncolytic virus. Vesicular stomatitis virus (VSV) is a negative-strand RNA virus that has a small genome encoding five proteins: nucleocapsid protein, phosphoprotein, matrix protein, glycoprotein and large polymerase protein [178]. Human VSV infections are usually asymptomatic, which poises VSV as a promising oncolytic therapeutic. VSV replication in host cells may generate copy-back defective interfering (DI) RNA, a truncated form of VSV genome. Both the defective interfering RNA and the whole genome of VSV may bind to RIG-I and thereby induce immune responses [179]. However, the immune responses to VSV infection may be compromised by the matrix protein of VSV, which targets the nucleoporin Nup98 and then inhibits nucleocytoplasmic trafficking of host cell mRNAs, thus suppressing the expression of host proteins including IFNs in infected cells [180, 181]. Given that some tumor cells may be resistant to VSV, novel VSV recombinants are needed for further development of VSV as effective therapeutics for cancer [182, 183]. VSV is currently being evaluated in phase I clinical trials against different malignancies.

Herpes simplex virus type 1 (HSV-1), a double-stranded linear DNA virus, is another oncolytic virus that has been used to treat cancer. Talimogene laherparepvec (T-VEC) is a HSV-1 recombinant with ICP34.5 and ICP47 deletion and GM-CSF insertion [184]. Intralesional immunotherapy with T-VEC has been approved for treating unresectable melanoma [185]. HSV-1 infection induces mitochondrial damage and mtDNA release, which triggers both cGAS/STING/IRF3 and RIG-I-MAVS signaling [186]. The induction of type I IFN expression by HSV-1 is largely dependent on RNA polymerase III, which catalyzes the synthesis of both mitochondrial and viral RNAs in the cytosol, thereby activating RIG-I [186]. In addition, HSV-1 infection leads to relocating the cellular *5S* rRNA pseudogene 141 transcripts that are recognized by RIG-I [187]. The small noncoding RNAs within HSV-1 latency-associated transcript also engage RIG-I to induce IFN expression [188]. Of note, the HSV-1 US11, US3 and UL31 γ134.5 proteins can suppress RIG-I signaling or IFN beta activity [189–191]. HSV-1 can evade the host innate immunity through complex mechanisms [192].

The dsRNA virus rotavirus strains can induce an MDA5-mediated immune response [193]. Intratumoral injection of rotavirus not only directly kills cancer cells but also upregulates the dsRNA sensors RIG-I and MDA5, stimulates type I IFN signaling, increases tumor-infiltrating myeloid cells, and activates tumor-infiltrative cytotoxic CD8^+^ T cells [194]. A preclinical study demonstrates that rotavirus has anticancer activities in vivo, synergizes with and overcomes anti–CTLA4 and anti-PD-L1 immunotherapy resistance [194]. Of note, inactivated rotavirus still upregulates RIG-I and synergizes with immune checkpoint blockade in tumor models, while it does not inhibit tumor when used as a monotherapy [194]. Thus, inactivated rotavirus may be prepared as a therapeutic cancer vaccine to revert “cold” tumors into immune-infiltrated “hot” tumors, and improve anti-PD1, anti-PD-L1 or anti-CTLA4 therapy. In addition, yellow-fever virus has oncolytic properties. Intratumoral injection of live attenuated yellow-fever vaccine induces type I IFN and promotes CD8^+^ T cells infiltration, thereby delaying tumor progression and enhancing the anticancer effects of anti-CD137 immunotherapy [195]. However, it remains unclear whether RIG-I and MDA5 are upregulated by yellow-fever virus, and to what extent RLRs contribute to yellow-fever virus-induced immune responses. It also remains to know whether inactivated or recombinant yellow-fever viruses can synergize with anti-CD137 therapy or other immune checkpoints blockade.

Newcastle disease virus (NDV) is another oncolytic virus being developed for cancer therapy. Upregulation of RIG-I was detected in cancer cells that were persistently infected with recombinant low-pathogenic NDV [196]. Activation of RIG-I by NDV may block the immune-suppressive effect of Treg cells [197]. Moreover, inactivated Sendai virus stimulates RIG-I and triggers antitumor immunity [198, 199]. Except for direct oncolysis, immune responses also contribute to the anticancer effects of reovirus, a naturally occurring and nonpathogenic dsRNA virus with oncolytic property [200]. Intratumoral reovirus synergizes with intravenous anti-PD1 to inhibit melanoma [201].

Vaccinia virus (VACV) is an oncolytic DNA virus under clinical testing. During VACV infection, RNA POLIII-mediated dsDNA-sensing pathway is activated, leading to the generation of dsRNA that engages RIG-1/MDA5 and TLR3 [202]. While the cellular actin nucleator Spire homolog 1 (Spir-1) can enhance RIG-I/MDA5 signaling [203], VACV proteins E3, D9 and D10 may prevent the accumulation of dsRNA or its sensing by RIG-I [204–206]. VACV recombinants with little restriction of host immune responses can be developed by genetic engineering. Deletion of selective VACV genes may allow tumor-selective replication and cytotoxicity [207, 208]. Recombinant VACVs have been developed as vaccine platforms for preventing infectious diseases and treating cancer. GM-CSF-armed VACV strains (JX-594/Pexa-Vec) have potent anticancer activity in preclinical models or cancer patients [209–211]. The modified vaccinia virus Ankara (MVA) is a highly attenuated vaccinia strain. MVA can be recognized by pattern recognition receptors including TLR3, RIG-I/MDA5, and cGAS/STING, thereby inducing apoptosis [212]. Interestingly, heat-inactivated MVA can induce higher levels of type I IFN in conventional dendritic cells and stronger antitumor immunity compared with live MVA [213]. Heat-inactivated MVA recombinant also generates stronger immunity and anticancer effect than a live counterpart when combined with anti-CTLA4 or anti-PD-L1 antibody in a murine melanoma model [214]. These data indicate that viral replication and viral-mediated oncolysis are not absolutely required for the antitumor activity of MVA. While intratumoral delivery of VACV can elicit antitumor immunity and tumor-suppressive effect, intravenous administration may be necessary for some clinical settings. A preclinical study indicates that intravenous injection of VACA strain JX-963 not only inhibits primary tumors but also suppresses distant metastases [210]. Another study suggests that pretreatment with PI3Kδ-selective inhibitors (IC87114 or idelalisib) may improve the intravenous delivery of VACV to tumors by inhibiting viral attachment to systemic macrophages, thus enhancing the antitumor efficacy [215]. The success in intravenous delivery of VACV to tumors or disseminating tumor cells may greatly improve the feasibility and efficacy of VACV therapy in clinical practice.

## Activation of RLR signaling for cancer therapy by viral mimicry

Except for exogenous RNA, endogenous small noncoding RNA can also engage RLRs to activate IFN production [216]. Around half of the mammalian genome is composed of transposable elements (TEs) such as DNA transposons and retrotransposons. TE-derived nuclei acids have a structure similar to viral nucleic acids. Therefore, the reactivation of TEs may trigger immune responses similar to viral infection [217]. The mammalian genomes contain retroelements such as long terminal DNA repeat (LTR), long interspersed nuclear DNA element (LINE), and short interspersed nuclear DNA element (SINE) flanking endogenous retroviral sequences (HERVs) [218]. While these viral sequences may remain transcriptionally silent in human genomes, derepression of these elements can be induced by epigenetic therapy. Viral mimicry is a cancer therapeutics that aims to awake epigenetically repressed viral genes and induce immune responses in tumors. DNA methylation inhibitors trigger the transcription of dsRNAs of repetitive elements from HERVs and thereby activate RIG-I and MDA5 [219]. LTR, intronic and intergenic SINE elements, and specifically inverted-repeat Alu elements are the major source of epigenetic therapy-induced immunogenic dsRNA [220, 221]. The hypomethylating agent decitabine has been approved for the treatment of myelodysplastic syndromes (MDS) and myelomonocytic leukemia [222]. However, the oral bioavailability of decitabine is compromised by cytidine deaminase in the gastrointestinal tract and liver [223]. This problem can be overcome by the cytidine deaminase inhibitor cedazuridine. Decitabine/cedazuridine received approval in the USA and Canada for treating MDS and chronic myelomonocytic leukemia [224]. The next-generation DNMT inhibitor guadecitabine is an investigational drug for treating MDS, AML and some solid tumors [225]. Recently, a reversible DNMT1-selective inhibitor has been developed with improved tolerability and efficacy in acute myeloid leukemia [226]. Furthermore, the combination of inhibitors of DNMT and ten–eleven translocation (TET) enzymes also increases the effects of viral mimicry featured by increased expression of ERV transcripts, cytosolic dsRNA, and activation of IFN response [227]. Inhibition of G9a/DNMT methyltransferase with CM-272 induces apoptosis and immunogenic cell death and suppresses HCC and cholangiocarcinoma [228–230].

Viral mimicry can also be induced by inhibitors of DNMT expression. RRx-001, a dinitroazetidine derivative, is an investigational anticancer agent that can inhibit DNMT1 and DNMT3A expression and mediates immunomodulatory effects [219]. Similar to 5-azacytidine, RRx-001 modulates antitumor immunity by increasing M1 macrophages [231]. In addition, epigenetic therapy induces ADAR1 dependency in cancer cells [232]. While epigenetic therapy reactivates repeat elements such as SINEs and Alu, inhibition of ADAR1 activity can stabilize inverted-repeat Alu dsRNA and reduce A to I editing of SINEs, leading to the recognition of Alu dsRNA and unedited SINEs by MDA5, followed by IFN-I response and inflammation [37, 220, 233]. These may explain why ADAR1 is required for the survival of cancer cells during epigenetic therapy. Meanwhile, the duration of endogenous retroviral element activation may be associated with the severity of inflammation resulting from ADAR1 inhibition.

Except for DNA methylation, histone methylation is another mechanism of epigenetic regulation of gene expression [234]. Dual inhibition of DNA and histone methyltransferases further enhances the anticancer effect of viral mimicry in ovarian cancer cells [235]. H3K9 methyltransferase (SETDB1) inhibition derepresses many transposable elements and activates RLRs signaling [236]. Protein arginine methyltransferase (PRMT) is another master epigenetic regulator and therapeutic target in cancer. The type I protein arginine methyltransferases (PRMTs) inhibitor MS203 induces dsRNA transcribed in part from inverted-repeat Alu elements and thereby activates interferon responses through the antiviral defense pathway, resulting in the inhibition of triple-negative breast cancer [237]. In addition, viral mimicry sensitizes melanoma to anti-PD1 and anti-CTLA4 therapy [238, 239]. However, not all viral-like sequences are tumor-suppressive. For example, human satellite II (HSATII) satellite repeat expression is negatively associated with IFN response and positively associated with a more aggressive phenotype in ovarian cancer [240].

## Activation of RLR signaling by radiotherapy, chemotherapy and molecular-targeted therapy

**Fig. 5:**
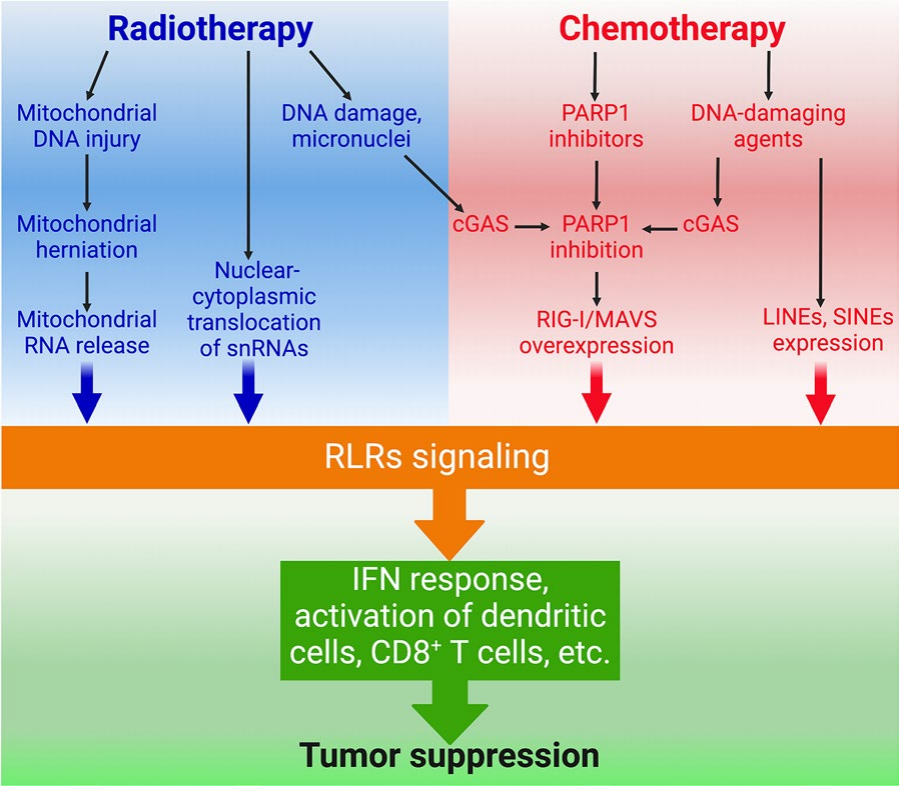
Mechanisms underlying the induction of RLRs signaling by radiotherapy and chemotherapy. Radiotherapy can induce mitochondrial DNA injury, the release of mitochondrial RNA into the cytosol and the nuclear–cytoplasmic translocation of small nucleolar RNAs (snRNAs), which engage RLRs activation. Both radiotherapy and DNA-damaging agents activate cGAS and thereby inhibit PARP1, leading to RIG-I/MAVS overexpression. DNA-damaging agents also induce long interspersed nuclear DNA element (LINE) and short interspersed nuclear DNA element (SINE) expression, thereby activating RLRs signaling

DNA damage and its repair defects are common in many cancer types. DNA damage repair defects lead to chromosome instability, which is a hallmark of cancer and a key mechanism of cancer development and progression [241]. Previous studies have demonstrated a negative correlation between DNA damage repair proteins and innate immune signaling [242, 243]. Ataxia–telangiectasia mutated (ATM) is a critical kinase in double-strand DNA repair. Loss of function mutations in ATM or the absence of ATM expression may trigger the release of DNA into the cytoplasm where it is recognized by STING and therefore induces IFNs expression [244]. In addition, depletion of the single-strand break repair protein PARP1 results in RIG-I/MAVS-mediated expression of interferon-stimulated genes [245, 246]. PARP1 depletion leads to overexpression of both RIG-I and MAVS, but it remains unclear what dsRNA species activate RIG-I under such circumstances [245]. Upon ionizing radiation, DNA damage and the formation of micronuclei initiate cGAS-STING-mediated IFN signaling [247, 248]. On the other hand, nuclear cGAS interacts with PARP1 and inhibits DNA repair [249]. It remains to know whether nuclear cGAS may promote RIG-I and MDA5 expression. In addition, DNA damage or genotoxic stress also activates SINEs and LINEs [250, 251], which engage RLRs to stimulate IFN signaling. RIG-I also reciprocally inhibits DNA repair by interacting with DNA repair factor XRCC4 and impairing its function [252]. Of note, the crosstalk between STING and MAVS is important for the full activation of cytoplasmic DNA- or RNA-induced IFN responses [253]. MAVS depletion suppresses the induction of TBK1 phosphorylation and IFN-β expression by cytoplasmic DNA [254]. Therefore, both the DNA and RNA sensing pathways are involved in DNA damage-induced immune responses. In addition, RLRs- and MAVS-dependent activation of IRF3 is critical for DNA double-strand breaks-induced cell death [255]. IRF3 may directly interact with pro-apoptotic factors and therefore promote apoptosis [256] (Fig. 5).

Radiation therapy is a standard-of-care treatment for many cancer types. Radiotherapy may not only induce cancer cell death but also increase intratumoral production of type I IFN, which promotes cross-priming of CD8^+^ T cells by tumor-infiltrating dendritic cells, thereby inducing T-cell-dependent tumor regression [257, 258]. However, activation of IFNAR1 in tumor cells by type I IFN may help them resist T lymphocytes and NK cells killing after radiation by upregulating Serpinb9 [259], which inhibits the cytotoxic molecule granzyme B secreted by T and NK cells [260]. Thus, inhibition of Serpinb9 and other pro-tumor elements downstream of IFN-I in tumor cells may enhance the efficacy of radiotherapy and immunotherapy. Preclinical studies indicate that the intratumoral immune activity and the lytic activity of CD8^+^ T cells are closely associated with the effectiveness of radiotherapy [261]. Both the cytoplasmic DNA and RNA sensing pathways are involved in the type I IFN responses after radiation therapy. Radiation therapy not only induces nuclear DNA damage but also triggers mitochondrial DNA injury, which promotes BAX-BAK-dependent mitochondrial herniation, thereby releasing both mitochondrial DNA and RNA into the cytosol [262]. However, the cytosolic mtDNA can be degraded by TREX1 nuclease, which may disable the DNA sensing pathway [262, 263]. Therefore, the levels of TREX1 or other negative regulators of DNA sensing may determine the extent to which STING signaling is activated after radiotherapy. On the other hand, the leaked mtRNA can engage RLR-MAVS signaling pathway to induce type I IFN responses after radiotherapy [262]. The leakage of mitochondrial RNA may be a generalized mechanism for activating RLRs under stress conditions, as many stressors could affect mitochondrial functioning and integrity.

Ionizing radiation also triggers the nuclear–cytoplasmic translocation of small nuclear RNAs including U1 and U2, which predominantly bind to RIG-I and induce IFN signaling after radiation [40]. Therefore, both mtDNA and snRNA may contribute to activating RIG-I and IFN signaling after radiation therapy. It warrants further studies to determine whether other endogenous RNAs are also involved in the activation of RIG-I after ionizing radiation. In addition, overexpression of LGP2 in cancer cells suppresses the induction of cell death and IFNβ expression by ionizing radiation [264], which may be attributable to the repression of RIG-I signaling by LGP2 [45–49]. However, LGP2 is required for dendritic cells to sense stimuli from irradiated tumor cells and produce type I IFN, and for their capability to prime T cells [265]. These data indicate that LGP2 is a contextual promoter or suppressor of radiation-induced IFN response. In fact, the context-dependent effects of LGP2 on RNA sensing and immune response have been demonstrated in multiple studies [48, 266, 267].

Of note, the effects of radiotherapy on the immune system are very complex. Radiotherapy may trigger both antitumor immune responses and immunosuppressive effects. The latter may be attributable to the upregulation of immune checkpoint molecules and expansion of immunosuppressive cells such as Treg cells and myeloid-derived suppressor cells [268]. The balance between the antitumor immunity and immunosuppressive response may determine the effectiveness of radiotherapy. Blockade of the immunosuppressive responses may improve radiotherapy or overcome radioresistance [269]. While there is evidence to suggest that anti-PD1/CTLA4 immune checkpoint inhibitors may enhance both local and distant tumor responses to radiotherapy in preclinical studies and some clinical trials [270, 271], the positive interaction between radiotherapy and immune checkpoint blockade is not achieved in many clinical settings [272]. It warrants further studies to determine how the synergy between radiotherapy and immune checkpoint blockade can be achieved in certain contexts. A recent study indicates that high tumor aneuploidy may be a biomarker for enhanced responsiveness to concurrent radiation and immune checkpoint blockade in patients with non-small cell lung cancer [272].

While radiotherapy has DNA-damaging effects, some chemotherapeutic agents also induce DNA damage. Treatment of cancer with DNA-damaging agents such as doxorubicin, etoposide, teniposide and oxaliplatin induces type I IFN responses and the activation of both dendritic cells and CD8^+^ T cells [40, 273, 274]. A recent study demonstrates that chemotherapy-induced transposable elements may activate MDA5 in hematopoietic stem cells to enable their exit from quiescence [275]. It warrants further study to determine how MDA5 is involved in the response of tumor and stromal cells to chemotherapy. In addition, recent studies indicate that RLRs signaling is involved in molecular-targeted therapy. The CDK inhibitor dinaciclib induces type I IFNs expression and synergizes with PD1 or PD-L1 blockade to inhibit cancer [276, 277]. Pyroptosis may mediate the induction of IFN response by CDK inhibitor [278]. It is unclear whether RLRs are involved in the induction of type I IFN response by dinaciclib. Moreover, EGFR inhibition triggers RIG-I-mediated type I IFN response in lung cancer, which, however, contributes to EGFR inhibitor resistance [279]. It remains to know whether targeting other oncogenes may induce RIG-I- or MDA5-mediated immune responses.

## Clinical testing of cancer therapy involving RLRs activation

Accumulating evidence from preclinical studies demonstrates that RLRs-targeted agents hold promise in cancer therapy. As described above, RLRs can be activated by small RNA, oncolytic viruses, viral mimicry and radio-chemotherapy. While dsRNA or stem-loop RNA is a direct agonist of RLRs, its delivery relies on synthetic polymers or nanoparticles. The clinical administration of these delivery systems is still limited. RIG-I agonists are still in the earliest phases of clinical testing for cancer therapy (Table 2). Little progresses have been achieved in treating cancer patients with synthetic RLRs agonists. Phase I studies of MK-4621, an oligonucleotide agonist of RIG-I, indicate that intratumoral injection of MK-4621/jetPEI™ or combination of MK-4621 with pembrolizumab (MK-3475; anti-PD1 mAb) is well tolerated and capable of activating RIG-I pathway in cancer patients, while no clinical benefit has been shown [280]. It is unclear whether the dose of MK-4621 should be adjusted, or systemic delivery of RLRs agonists may have different outcomes. In addition, a preclinical study demonstrates that combining systemic anti-PD1 therapy and intratumoral administration of CV8102, a cationic peptide-complexed ssRNA agonist of TLR7/8 and RIG-I, can induce antitumoral immune response [281]. A phase I study of intratumoral CV8102 delivery alone or in combination with systemic anti-PD1 therapy in patients with advanced melanoma, squamous cell carcinoma of the skin, head and neck, or adenoid cystic carcinoma is supposed to be completed soon (Table 2). Moreover, a phase I/II study of CV8102 and the therapeutic vaccine HepaVac-101 in treating HCC demonstrates that this treatment effectively induces immune responses [282]. While there are very limited clinical trials for systemic administration of 5′-pppRNA, multiple clinical trials have been conducted to determine the safety or efficacy of systemic administration of poly(I:C), a synthetic dsRNA mimic targeting TLR3 and MDA5. We can learn from the experience in intravenous or intramuscular administration of poly(I:C). Rintatolimod, Hiltonol and BO-112 are three poly(I:C)-based agents that have entered clinical development [283]. Hiltonol and BO-112 have been used safely for intratumoral, subcutaneous or intramuscular administration in cancer patients [284–287]. Of note, studies in mouse tumor model indicate that intratumoral injection of Hiltonol was substantially less effective compared to systemic delivery [288, 289]. Mechanistically, the superior antitumor effect of systemic delivery of Hiltonol may be due to the stimulation of MDA5 in bone marrow-derived immune cells and tumor vascular endothelial cells by Hiltonol, which leads to the production of type I IFN and T cell recruiting chemokines such as CXCL9/CXCL10, and the promotion of tumor T cell infiltration [289]. Systemic delivery of RLRs agonists may be considered in future clinical trials.

**Table 2:** Clinical trials of cancer therapy involving RLRs activation

Category	Agent	Combination	Disease	Phase	Outcome	Identifier	References
5′-pppRNA	MK-4621	Pembrolizumab	Solid tumors	I	OR with MK-4621 monotherapy, 0; SD, 4 (27%). PR with combination therapy, 3 (10%)	NCT03065023 NCT03739138	[280]
Synthetic dsRNA	BO-112	Nivolumab or pembrolizumab	Tumors that had primary resistance to anti-PD-1	I	PR and SD with combination therapy at 8–12 weeks, 3/28 (10.7%) and 10/28 (35.7%), respectively	NCT02828098	[287]
Peptide-complexed ssRNA with poly-U repeats	CV8102	HepaVac-101 (therapeutic vaccine)	HCC	I/II	Immune responses against ≥ 1 vaccinated HLA class I and II TAA, 37% and 53%, respectively	NCT03203005	[282]
ssRNA	CV8102	Anti-PD-1 therapy	Advanced melanoma; squamous cell carcinoma of the skin, head and neck; adenoid cystic cancer	I	Estimated study completion date, February 2023	NCT03291002	[281]
Oncolytic virus	T-VEC	Pembrolizumab	Advanced/metastatic sarcoma	II	Best ORR at 6 mo: 30%. ORR overall, 35%	NCT03069378	[291]
Oncolytic virus	VSV	Ruxolitinib phosphate	Advanced treatment-refractory T cell lymphoma	I	3/7 patients with T-cell lymphoma had responses: 2 PR at 3 mo and 6 mo, respectively; 1 CR ongoing at 20 mo	NCT03017820	[292]
Oncolytic virus	Pexa-Vec/ JX-594	Metronomic cyclophosphamide	Advanced/metastatic soft tissue sarcoma	II	Combination therapy is not superior to metronomic cyclophosphamide alone (median PFS, 1.7 mo vs 7 mo; OS, 14.2 mo vs not reached	NCT02630368	[297]
Oncolytic virus	Pexa-Vec	Avelumab	Soft tissue sarcoma	I/II	Ongoing	NCT02630368	/
Oncolytic virus	Pexa-Vec	Surgical treatment	Colorectal cancer liver metastases or metastatic melanoma	II	Presurgical treatment with Pexa-Vec was associated with IFNα and chemokine induction, resulting in transient innate and long-lived adaptive anticancer immunity	EudraCT number 2012–000,704-15	[298]
Oncolytic virus	Pelareorep (reovirus)	Paclitaxel	Metastatic breast cancer	II	Median adjusted PFS (combination therapy vs paclitaxel alone), 3.78 mo vs 3.38 mo; Median OS (combination therapy vs paclitaxel alone), 17.4 mo vs 10.4 mo	NCT01656538	[303]
Oncolytic virus	Pelareorep	Pembrolizumab, and either 5-fluorouracil, gemcitabine, or irinotecan	Advanced pancreatic adenocarcinoma	Ib	Partial response for 17.4 mo, 1/10; SD, 2/10, lasting 9 and 4 mo, respectively	NCT02620423	[305]
Viral mimicry	Azacitidine	Nivolumab	Relapsed/refractory AML	Ib/II	ORR,33%; CR, 22%; 1 PR, 10% with hematologic improvement maintained > 6 mo. SD (> 6 mo), 10%; ORR was 58% and 22%, in hypomethylating agent-naïve and HMA-pretreated patients, respectively	NCT02397720	[307]
Viral mimicry	Azacitidine	Camrelizumab	Relapsed/refractory classical Hodgkin lymphoma	II	CR, 79% in the decitabine-plus-camrelizumab group vs 32% in camrelizumab group. Median PFS with decitabine-plus-camrelizumab therapy, 35.0 mo; 15.5 mo with camrelizumab monotherapy	NCT02961101 NCT03250962	[311]
Viral mimicry	Decitabine	Camrelizumab	Relapsed/refractory classical Hodgkin lymphoma after prior anti-PD-1 monotherapy	II	ORR, 52%; CR, 36% in the test cohort. ORR, 68%; CR, 24% in the expansion cohort. Median PFS in the test cohort and expansion cohort, 20 and 21.6 mo, respectively	NCT02961101 NCT03250962	[312]
Viral mimicry	Guadecitabine	Pembrolizumab	Advanced solid tumors	I	ORR, 7% with 37% achieving disease control (PFS) for ≥ 24 weeks. 5/12 (42%) NSCLC patients have disease control ≥ 24 weeks	NCT02998567	[314]
Viral mimicry	Entinostat	Pembrolizumab	Metastatic uveal melanoma	II	ORR,14%. CBR 18 weeks, 28%; median PFS2.1 months; median OS, 13.4 months. Toxicities were manageable, and there were no treatment-related deaths	NCT02697630	[316]
Chemotherapy	Capecitabine and oxaliplatin	Pembrolizumab	Advanced biliary tract carcinoma	II	PR, 27.3%; SD, 54%. Disease control rate, 81.8%. Median PFS, 4.1 mo with a 6 mo PFS rate of 45.5%	NCT03111732	[319]

CR, complete response; mo, months; OR, overall response; ORR, objective response rate; OS, overall survival; PR, partial response; PFS, progression-free survival; SD, stable disease. The identifier is the registration number in clinicaltrials.gov unless otherwise indicated

Since the US FDA approved the first oncolytic virus drug talimogene laherparepvec (T-VEC, IMLYGIC) in 2015, there are much oncolytic virotherapy that has been evaluated in clinical trials [290, 291]. Given that dsRNA is the major ligand of RLRs, this review will focus on oncolytic RNA viruses that can activate RLRs signaling. Intravenous delivery of VSV armed with IFN-β in 15 patients with relapsed refractory hematological malignancies has no dose-limiting toxicities and elicits encouraging dose-dependent efficacy among patients with advanced treatment-refractory T cell lymphoma [292]. To further relieve the neurotropism of VSV, a recombinant VSV (VSV-GP) with the substitution of its neurotropic glycoprotein G into the non-neurotropic GP of the lymphocytic choriomeningitis virus has been developed [293, 294]. Preclinical studies show that both intratumoral and intravenous delivery of this recombinant VSV can effectively inhibit tumor growth and metastasis [295]. A phase I clinical trial has been initiated to evaluate the safety and early efficacy of intratumoral or intravenous delivery of VSV-GP alone or in combination with the immune checkpoint inhibitor ezabenlimab [296]. Pexa-Vec (JX-594) is another recombinant VSV with deletion of thymidine kinase gene, which attenuates VSV replication in tumor tissue [297]. Presurgical intravenous infusion of Pexa-Vec may stimulate anticancer immunity and treat patients with cancer metastasis [298]. It warrants further studies to determine if inactivated VSV can synergize with an immune checkpoint inhibitor to treat cancer patients.

Previous clinical trials have demonstrated that the naturally occurring reovirus type 3 Dearing and the nongenetically modified serotype 3 reovirus pelareorep can be safely combined with conventional chemotherapy in patients with advanced cancer [299–301]. In 34 chemotherapy-naïve patients with advanced pancreatic adenocarcinoma, intravenous delivery of pelareorep and gemcitabine triggers a partial response in one patient and stabilizes disease in 23 patients [302]. A phase II, randomized study of pelareorep and paclitaxel in previously treated and metastatic breast cancer showed a significantly longer overall survival for this combination, while there was no difference in progression-free survival [303]. However, randomized phase II trials of pelareorep–paclitaxel combination in patients with pretreated, advanced or metastatic non-small cell lung cancer or patients with untreated metastatic pancreatic adenocarcinoma did not show improved progression-free survival [301, 304]. With regard to pelareorep in combination with an immune checkpoint inhibitor, a phase Ib trial shows that pelareorep–pembrolizumab combination in patients with pancreatic adenocarcinoma is well tolerated and has prolonged efficacy in some patients [305]. A follow-up phase II study with pelareorep and pembrolizumab as a second-line treatment for pancreatic adenocarcinoma is underway (NCT03723915). Since a previous study has indicated that r*eovirus* replication is not required for the generation of human antitumor immunity, it warrants further studies to determine whether intravenous delivery of inactivated reovirus can also prime “immune-cold” tumors for response to immune checkpoint blockade.

With regard to viral mimicry, randomized phase III trial in patients with acute myeloid leukemia demonstrates that oral azacitidine maintenance has a generally favorable safety profile [306]. The combination of viral mimicry with immune checkpoint blockade has been extensively evaluated in clinical trials. Phase II clinical trial demonstrates that azacitidine in combination with nivolumab has well-tolerable safety, while treatment-related adverse events include neutropenia, anemia and immune-related adverse events such as pneumonitis [307]. Azacitidine and nivolumab combination appears to be an effective therapy for relapsed or refractory AML, especially for patients who were salvage 1, prior hypomethylating agent-naïve, or had increased pretherapy CD3^+^ bone marrow infiltrate [307]. Another phase Ib/II study of azacitidine and PD-L1 antibody avelumab in relapsed/refractory AML suggests that this treatment does not confer clinical benefit, possibly due to overexpression of PD-L2 in these patients and the low percentage of hypomethylator-naïve subjects [308]. A pilot study of decitabine and PD1 antibody pembrolizumab in adult patients with refractory/relapsed AML shows the best response of stable disease or better in 6 of 10 patients [309]. However, a phase II clinical study indicates that pembrolizumab and azacitidine combination confers modest clinical activity in treating chemotherapy-refractory metastatic colorectal cancer [310]. In addition, phase II study of decitabine and PD1 antibody camrelizumab in relapsed/refractory classical Hodgkin lymphoma shows that decitabine–camrelizumab combination has increased effectiveness compared with camelizumab monotherapy [311]. Even though classical Hodgkin lymphoma patients relapsed after prior camrelizumab monotherapy, a combination of decitabine and camrelizumab was still associated with high response rates and improvement in progression-free survival [312]. Based on these promising results, phase III clinical trials of decitabine and camelizumab or talacotuzumab in AML and Hodgkin lymphoma have been initiated. Moreover, combining the next-generation hypomethylator guadecitabine and ipilimumab is safe and tolerable in patients with unresectable melanoma, and has promising immunomodulatory and antitumor activity [313]. Another phase I trial also shows that guadecitabine in combination with pembrolizumab is tolerable with immunomodulatory and anticancer activity in patients with advanced solid tumors, MDS or AML [314]. Reversal of previous resistance to immune checkpoint inhibitors is demonstrated in this study [314].

The combination of HDAC inhibitors such as vorinostat with pembrolizumab is being tested in patients with breast cancer. Pembrolizumab–vorinostat combination is well tolerated and has preliminary antitumor activity despite progression on prior ICI treatment in patients with advanced/metastatic non-small cell lung cancer [315]. Another phase II study indicates that combining the HDAC inhibitor entinostat with pembrolizumab confers durable responses in a subset of patients with metastatic uveal melanoma [316]. In addition, treatment of PD-L1 antibody-resistant/refractory NSCLC patients with pembrolizumab plus entinostat produces a clinically meaningful benefit, with objective response in 9% of patients [317]. The levels of circulating classical monocytes at baseline may be a potential biomarker for response to this regimen [317]. No phase III clinical trial of HDAC inhibition in combination with immune checkpoint blockade in the treatment of cancer has been registered.

Since radio-chemotherapy-induced DNA damage may activate RLRs signaling and induce an immune response, the combination of radio-chemotherapy with immune checkpoint inhibitors has been evaluated in clinical trials [318, 319]. The randomized PACIFIC study demonstrates that treatment of stage III NSCLC patients, who do not have disease progression after platinum-based chemoradiotherapy, with anti-PD-L1 antibody durvalumab improves the overall survival [271]. The randomized PEMBRO-RT trial shows that treatment of NSCLC patients with pembrolizumab after 3 fractions of 8 Gy radiotherapy increases the response rates and median survival [320]. Another trial in castration-resistant prostate cancer patients shows that a combination of radiotherapy with ipilimumab immunotherapy significantly increases overall survival rates compared with patients receiving ipilimumab only [321]. In addition, the preliminary data from a phase II trial indicate that radiation therapy may enhance the response to immune checkpoint blockade in microsatellite-stable colorectal and pancreatic adenocarcinoma [322]. The extent to which RLRs are involved in the response to radio-immunotherapy remains to be defined.

While there may be an advantage for combined treatment with radiotherapy and immune checkpoint blockade in some types of cancer, some clinical trials fail to show a superior effect of radiotherapy in combination with immune checkpoint blockade compared with radiotherapy plus chemotherapy or molecular-targeted therapy [323–326]**.** Treatment of patients with locally advanced-squamous cell carcinoma of head and neck by pembrolizumab in combination with radiotherapy fails to improve the tumor control and survival compared with the cetuximab (anti-EGFR monoclonal antibody)-radiotherapy arm, while the toxicity appears to be less in the pembrolizumab–radiotherapy arm [326]. A randomized phase III trial in patients with glioblastoma harboring unmethylated methylguanine-DNA methyltransferase (MGMT) promoter showed that the median overall survival of patients treated with standard radiotherapy and nivolumab is shorter than that in patients treated with radiotherapy and temozolomide [324]. Another phase III randomized CheckMate 548 study in patients with newly diagnosed glioblastoma with methylated or indeterminate MGMT promoter demonstrates that nivolumab in combination with radiotherapy and temozolomide does not improve the progression-free survival and overall survival compared with the standard treatment (radiotherapy plus temozolomide) [325]. In contrast, a phase II, nonrandomized study indicates promising antitumor activity of pembrolizumab in combination with concurrent chemoradiation therapy in patients with treatment-naïve, locally advanced, stage III non-small cell lung cancer [319]. The identification of predictive biomarkers and the timing of radiotherapy and immune checkpoint blockade may be important to allow a subset of cancer patients to benefit from combination therapy [272].

## Conclusions and perspectives

RLRs have vital roles in host immunity against pathogen infection. During host–microbe interaction, pathogens may escape from the host innate immunity by disabling the RLRs signal pathways. Defective host immunity leads to chronic infection, tissue damage and carcinogenesis. Downregulation of RLRs is either positively or negatively associated with the prognosis of different types of cancer. Preclinical studies have demonstrated that stimulation of RLRs signaling could induce immunogenic cell death and sensitize some types of “immune cold" tumors to immune checkpoint blockers. In fact, RLRs signaling is involved in established cancer therapies including oncolytic virus therapy, viral mimicry and radio-chemotherapy. While preclinical studies have shown that direct stimulation of RLRs by their agonists can inhibit some types of cancer, especially when it is combined with immune checkpoint blockade, such treatment has remained a proof-of-concept and has not been validated in late-phase clinical trials. We may keep a close eye on clinical testing of the anticancer effects of bifunctional 5′-ppp siRNA.

While RIG-I has critical roles in innate immune response, it also interacts with oncoproteins or tumor suppressors and then provokes non-immune functions. RIG-I may promote STAT1 activation, thereby inhibiting leukemia cell proliferation [325]. In addition, RIG-I binds to the non-receptor tyrosine kinase Src and inhibits Akt phosphorylation [326]. Given that Akt promotes tumorigenesis [327], the inhibition of Akt by RIG-I may be attributable to its tumor-suppressive effects. However, RIG-I also promotes STAT1-mediated upregulation of Notch targets and abrogates AMPK-mediated suppression of lipid synthesis, which contributes to the positive regulation of drug resistance and tumorigenesis by RIG-I in some contexts [328, 329]. Hence, activation of RIG-I in non-immune or cancer cells may have detrimental effects in a context-dependent manner. Likewise, many studies suggest that the induction of IFN, a crucial RLRs downstream effector, in tumor cells may be detrimental, indicating that strategies to target RLRs activation in immune cells rather than tumor cells may be required to improve the anticancer efficacy [330–332]. The specific delivery of RNA to immune cells can be achieved by modified lipid nanoparticles. It warrants further studies to determine whether the new generation of lipid nanoparticles could enable the RLRs-targeting RNA to effectively treat cancer. In addition, delivery of RLRs-activating RNA to immune cells in the tumor microenvironment by CAR-T cells is a promising strategy, while it remains to be evaluated in the clinical setting. The recruitment of CAR-T cells into tumors and the complex tumor microenvironment that prevent treatment success of CAR-T cells in many types of tumors may still be the bottleneck of this therapeutic option. Shuttling the RNA agonists of RLRs via immune cells-targeting exosomes or nanoparticles warrants further studies.

RIG-I and MDA5 have a preference for different RNA species, while they also respond to overlapping ligands. Whether RIG-I or MDA5 ligands should be exploited to treat cancer may depend on the elements in each axis. The exploitation of RLRs agonists for cancer therapy may need to be tailored by biomarkers such as key elements in RLRs signaling. Because many pathogens have evolved mechanisms of inactivating host innate immunity, loss or downregulation of RLRs and their effectors may be more common in infection-related cancer compared with non-infection-related cancer such as breast cancer, ovarian cancer and melanoma, suggesting potentially wider applicability of therapeutic RIG-I/MDA5 agonists in cancers that are not associated with chronic infection. If both RIG-I and MDA5 are impaired, targeting the downstream effector IRF3 by a small molecule activator may be a preferred choice [333].

While hypomethylating agents and oncolytic viruses can trigger RLRs signaling, they have many pleiotropic effects independent of RLRs. In fact, the success of cancer therapy largely relies on combination strategies [334]. Oncolytic viruses can also serve as vectors for other therapeutic agents and synergize with other immunotherapies [177]. Compared with synthetic RLRs agonists, oncolytic viruses and viral mimicry may have more profound anticancer effects, especially when they are combined with other immunotherapies. Of note, cancer immunotherapy may be limited by the toxicity triggered by the systemic delivery of potent immunomodulators. Given that inactivated oncolytic RNA viruses can trigger RLRs signaling and sensitize tumor cells to immune checkpoint blockade, future clinical trials are warranted to evaluate the anticancer effects of systemic delivery of inactivated oncolytic RNA viruses and immune checkpoint inhibitors. Similar to chemoresistance and radioresistance, the resistance to cancer immunotherapy is also a critical challenge. The mechanisms of RLRs signaling and their roles in immune responses are complex. Combination strategies to mitigate the paradoxical effects of RLRs in cancer cells, and the limitations of resistance to radiotherapy, chemotherapy and immunotherapy may be necessary to empower RLRs activation for cancer therapy.

## Abbreviations

ADAR1: Adenosine deaminase acting on RNA 1
cGAS: Cyclic GMP-AMP synthase
circular RNA: CircRNA
DAMPs: Damage-associated molecular patterns
EBV: Epstein–Barr virus
EVs: Extracellular vesicles
H. Pylori: Helicobacter pylori
HBV: Hepatitis B virus
HCV: Hepatitis C virus
HPV: Human papillomavirus
ICI: Immune checkpoint inhibitors
ISGs: Interferon-stimulated genes
LCP: Lipid–calcium–phosphate
LGP2: Laboratory of genetics and physiology 2
LNP: Lipid nanoparticle
LINE: Long interspersed nuclear DNA element
MAVS: Mitochondrial antiviral signaling protein
MDA5: Melanoma differentiation-associated gene 5
NDR2: Nuclear Dbf2-related kinase 2
NLRP: Nod-like receptor family pyrin domain-containing protein
NOD2: Nucleotide-binding oligomerization domain-containing protein 2
PAMPs: Pathogen-associated molecular patterns
PLGA: Poly(lactic-*co*-glycolic acid)
PRRs: Pattern-recognition receptors
poly I:C: Polyriboinosinic-polyribocytidylic acid
RIG-I: Retinoic acid-inducible gene 1
RLRs: RIG-I-like receptors
RIOK3: RIO kinase 3
RNAPOLIII: RNA polymerase III
snRNA: Small nucleolar RNA
srpRNA: Signal recognition particle RNA
SINE: Short interspersed nuclear DNA element
STING: Stimulator of interferon response CGAMP interactor
TLRs: Toll-like receptors
